# High-resolution transcript profiling reveals shoot abscission process of spruce dwarf mistletoe *Arceuthobium sichuanense* in response to ethephon

**DOI:** 10.1038/srep38889

**Published:** 2016-12-12

**Authors:** Yonglin Wang, Dianguang Xiong, Ning Jiang, Xuewu Li, Qiqing Yang, Chengming Tian

**Affiliations:** 1 The Key Laboratory for Silviculture and Conservation of Ministry of Education, College of Forestry, Beijing Forestry University, Beijing, China; 2 Academy of Forest Inventory and Planning, State Forestry Administration, Beijing, China; 3 Forest Pest Control and Quarantine Station of Qinghai Province, Xining, China

## Abstract

*Arceuthobium* (dwarf mistletoes) are hemiparasites that may cause great damage to infected trees belonging to Pinaceae and Cupressaceae. Currently, dwarf mistletoe control involves the use of the ethylene-producing product ethephon (ETH), which acts by inducing dwarf mistletoe shoot abscission. However, the process by which ETH functions is mostly unknown. Therefore, the transcriptome of the ETH-exposed plants was compared to non-exposed controls to identify genes associated with the response to ethephon. In this study, the reference transcriptome was contained 120,316 annotated unigenes, with a total of 21,764 ETH-responsive differentially expressed unigenes were identified. These ETH-associated genes clustered into 20 distinctly expressed pattern groups, providing a view of molecular events with good spatial and temporal resolution. As expected, the greatest number of unigenes with changed expression were observed at the onset of abscission, suggesting induction by ethylene. ETH also affected genes associated with shoot abscission processes including hormone biosynthesis and signaling, cell wall hydrolysis and modification, lipid transference, and more. The comprehensive transcriptome data set provides a wealth of genomic resources for dwarf mistletoe communities and contributes to a better understanding of the molecular regulatory mechanism of ethylene-caused shoots abscission.

Conifers, found abundantly in the Northern Hemisphere, are of huge ecological and economic values and serve as key species in many other ecosystems. However, pests and plant diseases are a major threat to these trees. A main threat to conifer are parasitic flowering plants called heterotrophic plants. These plants acquire water and nutrients and establish vascular connections with the host plant.

The genus Arceuthobium (Family: Viscaceae) is a clearly defined group of small (generally less than 20 cm high) flowering plants called dwarf mistletoes, and are obligate heterotrophic plants that parasitize members of Pinaceae and Cupressaceae^1^,^2^. They are considered to be the most evolutionarily specialized genus of the family Viscaceae. Conifers infected with dwarf mistletoes exhibit large “witches’ brooms” and can be killed either directly or by rendering them more susceptible to insects or other pathogens.

*Arceuthobium sichuanense* (commonly known as spruce dwarf mistletoe, SDM) is a Chinese endemic plant parasite and the most serious vascular parasite of *Picea* and *Pinus* in China. During parasitism, the SDM endophytic system, referred to as the haustoria and bark strands, grow and develop within the spruce branch^1^,^3^. Once the infection is established, an incubation period of 2 to 5 years elapses before the young shoots appear^1^,^3^. SDM causes serious mortality in both mature and young spruce trees and has a severe impact on ecological safety in the Sanjiangyuan area of Qinghai province^4^. Despite the great economic and ecological importance of SDM in China, little is known about the basic mechanisms underlying its development and controls. With the increasing recognition of dwarf mistletoes as devastating parasites, the reduction of the damage caused by such parasites depends on adequate knowledge of *Arceuthobium*, indicating an increased need for studies on the members of the group.

Due to the extensive timber and ecological loss caused by dwarf mistletoes in coniferous forests, considerable efforts have been made to control them. Clearcutting as a feasible silvicultural control is an effective way to control them^1^. However, the control of dwarf mistletoes is complex and difficult in practice. Chemical control using herbicides has limited success, mainly because it is expensive and difficult to find chemical agents that affect only the mistletoe and not the host. Herbicides that kill the parasite, including the endophytic system, usually damage the host as well. One management option for dwarf mistletoes is the application of ethephon (ETH, ethylene-releasing growth regulator), which can induce abscission of dwarf mistletoe shoots. However, the chemical does not affect the endophytic system. Thus, the infected trees will need to be resprayed with the chemical every few years when new dwarf mistletoe shoots appear so as to prevent the production of inoculum^5^. Studies have reported the successful use of ethephon for controlling *Arceuthobium pusillum* on *Picea mariana* in Minnesota and *A. sichuanense* on *Picea crassifolia* in Qinghai^1^,^3^. Because of the success of ETH as a management tool, understanding the regulatory effects of ETH on dwarf mistletoe shoot abscission is of considerable value for disease management. However, little is known about the morphological events and molecular pathways involved in dwarf mistletoe shoot abscission after ETH application.

This study aims to understand the molecular pathways of SDM abscission by examining changes in the transcriptome over time using RNA-Seq. RNA-Seq is a developed approach for transcriptome profiling and provides a far more precise measurement of the levels of transcripts, which generates absolute, rather than relative, gene expression measurements^6^. RNA-Seq technology, accompanied by sophisticated bioinformatics tools for data analysis, including high performance de novo transcriptome assembly, facilitate transcriptome analysis in uncharacterized model plants, especially in parasitic plants including agricultural weed (*Ipomoea purpurea*), weed dodder (*Cuscuta pentagona*) and mistletoe (*Viscum album*)^7^,^8^,^9^. Thus, this study monitored the physiological and molecular status of SDM through a progressive experiment and characterized the temporal response of the transcriptome. One, three, six, nine, and twelve days after ETH application changes in the transcriptome were observed to characterize the changes in gene expression during the different steps that make up the abscission process.

## Results

### Effects of ETH on shoots abscission of SDM, protein levels, and plant hormones

**Figure 1:**
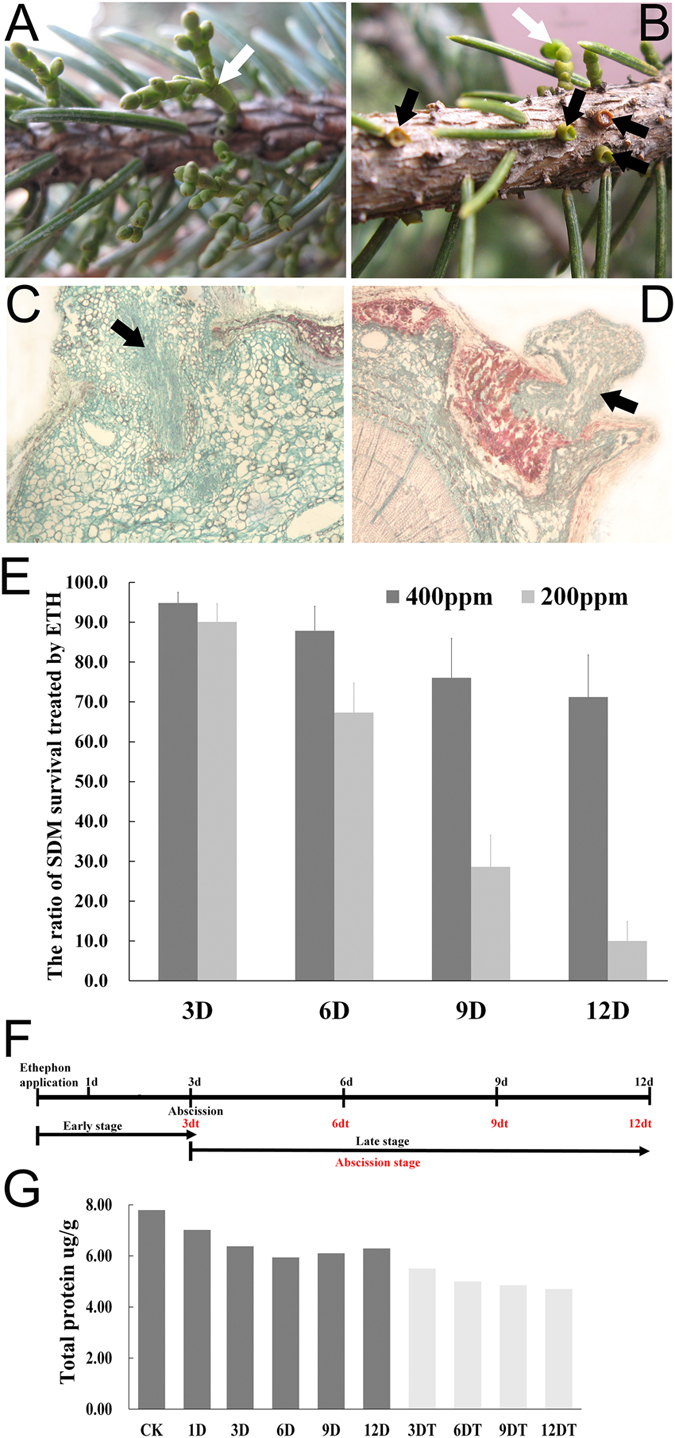
Effects of ethephon treatment on *Arceuthobium*. (**A** and **B**) The abscission of SDM shoots at 3 days after water (**A**) and ethephon (**B**) treatment. Black arrows represent abscission sites of SDM shoots and white arrow shows non-abscised DM shoots. (**C** and **D**) Longitudinal section of SDM shoots emerging from spruce braches. Black arrows represent SDM shoots colonizing in the spruce braches. Blue and red portion show the living and dead plant cells of SDM and spruce. (**E**) SDM survival after ethephon application at different concentrations (200 and 400 ppm) during time course. (**F**) Schematic representation of the experimental design. SDM shoots were treated with 200 ppm ethephon. Abscised and non-abscised DM shoots were collected at early (1 and 3 days), late (6, 9 and 12 days), and abscised stages (3dt-12dt in red). (**G**) Total protein levels were measured in SDM shoots during time course.

ETH-treated SDM showed a striking acceleration of the shoots abscission compared with the water-treated control (Fig. 1A, B). Observation of longitudinal section of SDM shoots emerging from spruce branch demonstrated that ETH induced cell death in the cortex of SDM and spruce, resulting in the shoot abscission (Fig. 1C,D). Overall, 200 ppm ETH caused higher abscission ratio that 400 ppm ETH. The ETH-induced abscission started as soon as 72 h after the treatment and was increasing even up to 12 days after the beginning of the treatment (Fig. 1E). In this study, samples of SDM shoots were collected at 1, 3, 6, 9, and 12 days after ETH treatment and used to compare the gene expression profile of non-abscised and abscised shoots (Fig. 1F).

The relative protein levels were observed to drop at 1 day, indicating that some proteins degraded during the ETH treatment. Furthermore, the relative protein levels of non-abscised shoots were higher than the abscised shoots (Fig. 1G).

**Figure 2:**
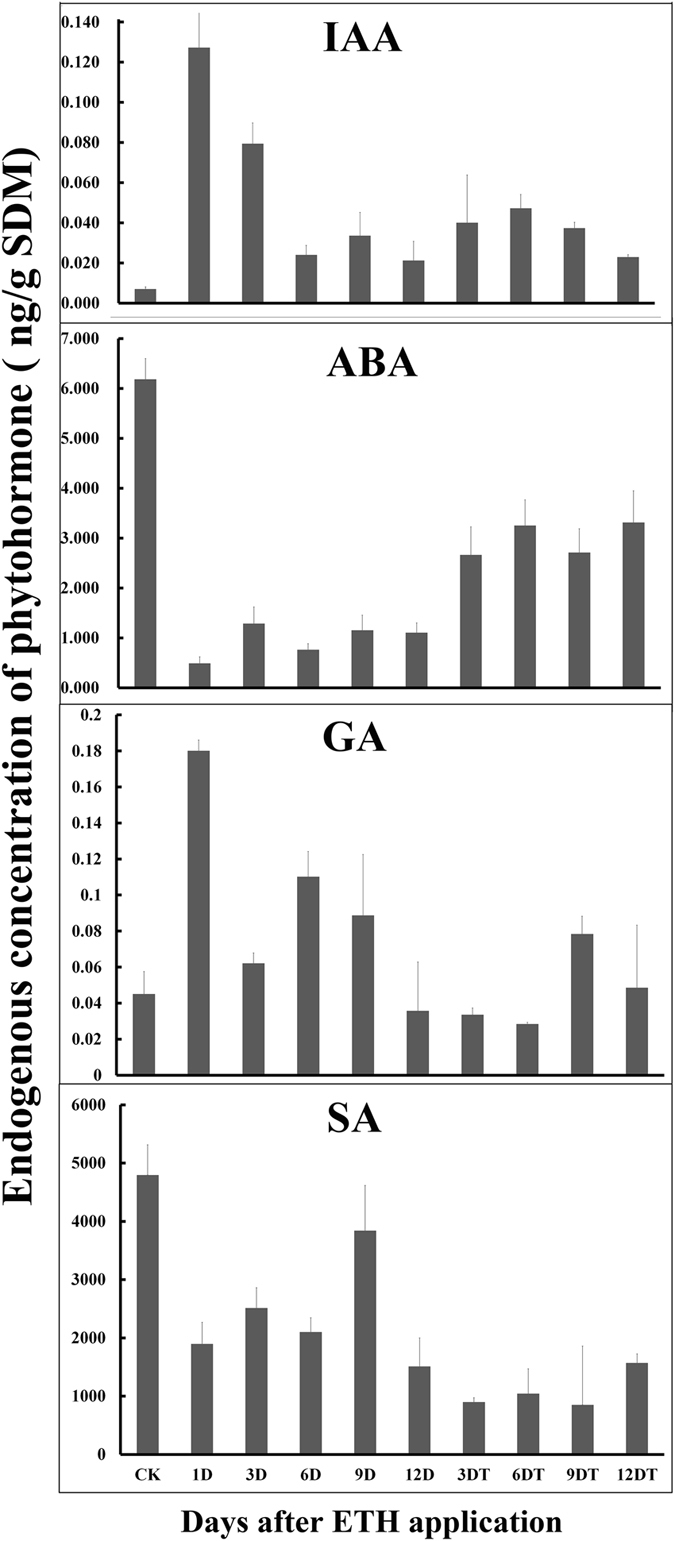
Hormone levels during ethephon treatment. Levels of IAA, ABA, GA and SA were measured in SDM shoots during time course. Values represent the mean of four independent biological replicates per time point. Bars represent standard errors.

Levels of the plant hormones indole acetic acid (IAA), abscisic acid (ABA), Gibberellin (GA), salicylic acid (SA) were quantified. IAA and GA were found to be significantly increased to the maximum at 1 day and then gradually declined. The levels of IAA in abscised shoots were higher than in non-abscised shoots while the opposite trend was observed in SA levels, i.e., the SA level in non-abscised shoots was higher (Fig. 2). ABA levels did not differ due to ETH treatments. The results suggested that ETH-induced abscission of SDM shoots resulted in biochemical changes including proteins and phytohormones.

### De novo assembly and annotation of SDM transcriptome

Generating a reference transcriptome with sufficient depth coverage was required to carry out differential gene expression analysis for SDM. Initially, massive Illumina short-reads were generated from libraries of the non-abscised and abscised shoots (Table 1). A total of 90,823,070 paired-end raw reads were obtained after sequencing libraries on the Illumina HiSeq2000 platform. After removing low-quality sequence, duplicated reads, and reads containing adapter/primer sequences, more than 88 million high-quality paired-end reads were used to assemble the SDM transcriptome. The resulting SDM transcriptome with 120,316 unigenes was subsequently used for downstream differential expression analysis (Table 2). The de novo assembly generated a high number of transcripts particularly enriched in small size unigenes with approximately 67% of unigenes are in the size range 200–500 bp (Supplemental Fig. S1). The reads used to assemble the transcriptome were mapped to the final transcriptome, and approximately 83% reads were perfectly mapped back.

To evaluate the quality of the SDM final transcriptome, the number of unigenes annotated by each database is summarized in Table 3. Notably, of the 120,316 assembled unigenes, 56,487 (46.95%) exhibited sequence homology to a sequence within the NCBI NR database; 62,736 (52.14%) unigenes were annotated in at least one database (Table 3; Supplemental Dataset 1). Moreover, 47.85% of the unigenes could not be identified, which is common in de novo sequencing studies. On the other hand, the prediction of likely coding sequences from 120,316 assembled unigenes resulted in 55,153 putative open reading frames (ORFs)/coding sequences.

**Figure 3:**
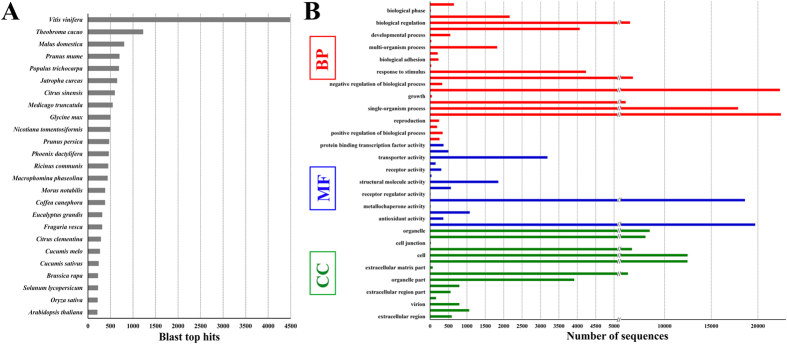
Annotation of the SDM transcriptome. (**A**) Top 20 species distribution of the SDM transcriptome showing the abundance of sequences. (**B**) Go category distribution of SDM transcripts among level 1 GO categories: biological process (BP), molecular function (MF), and cellular component (CC).

BLASTX against the NR database provided insight into the taxonomic distribution of the unigenes (Fig. 3A). More than 4,487 of the unigenes had top hits to sequences from *Vitis vinifera*. Only a few top hits were found from different species. Among the 120,316 unigenes, 96868 unigenes (80.51%), 61813 unigenes (51.37% of), and 46754 unigenes (38.86%) were assigned to GO terms. Multilevel GO distribution within these broad GO categories is shown in Fig. 3B and Supplemental Dataset 2. In addition, all unigenes were annotated as enzymes and were confirmed by the Enzyme Code number provided by KEGG in Supplemental Dataset 3.

### Global analysis of ETH-induced shoots transcriptome

To elucidate the regulatory network of SDM shoot abscission triggered by ethylene, 20 paired-libraries covering 6 different time points of non-abscised and abscised SDM shoots were produced by RNA-Seq technique. After quality filtering, 10–14 million reads for each library were generated from the above libraries (Table 1). For each RNA-Seq library, more than 88% of clean reads were mapped to the SDM reference transcriptome (Table 1).

**Figure 4:**
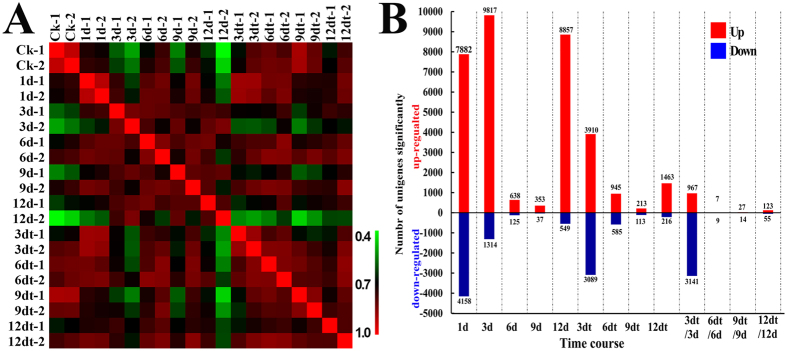
Correlation and profiling of transcriptomic response to ETH using RNA-Seq. (**A**) Correlation matrix of the whole data set. The analysis was performed by comparing the values of the entire transcriptome (120316 unigenes) in all 22 samples with two biological replicates. Correlation analysis (coefficients R^2^) was performed using R software. (**B**) Number of significantly differentially expressed genes after ETH treatment (P < 0.05, FDR < 0.05).

We found that distributions of gene expression levels were comparable irrespective of the sample type (Supplemental Fig. S2; Supplemental Dataset 4). The gene expression correlations between two biological replicates were high coefficients (R^2^ > 0.86) (Fig. 4A), demonstrating the reliability of the data produced and illustrating the consistency of the transcriptional changes within each sample. Genes differentially expressed among libraries (FDR < 0.05) were defined. Differential expression analysis showed that, of the 120,316 SDM unigenes, a total of 21,764 unigenes were significantly differentially expressed in the conditions analyzed (FDR < 0.05). The greatest number of unigenes with changed expression was found at the onset of abscission (3 days) induced by ETH. Increased expression of unigenes at 3 days was statistically significant (Fig. 4B). In addition, Fig. 4B suggested that the number of differentially expressed genes varied during the abscission process. We randomly selected genes that were analyzed by quantitative real-time PCR to verify their expression patterns (data not shown).

**Figure 5:**
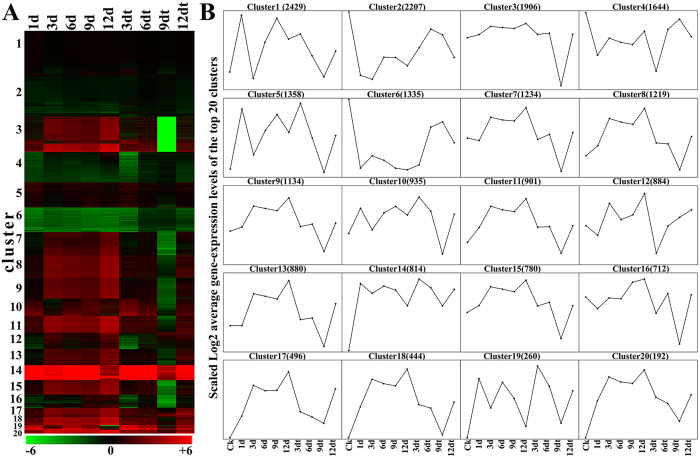
Clustering of the transcriptome response to ethephon. (**A**) Heatmap showing 21,764 unigenes expression clusters generated by the clustering affinity search technique method. Each line refers to data of one gene. The order is from the cluster with the most members (2,429 genes) to that with the least members (192). The color bar represents the log2 of FPKM values, ranging from green (−6), black (0) to red (6.0). (**B**) Log2 average gene-expression levels of the top 20 clusters. The number of cluster members with the number of associated unigenes in parentheses was on the top of the plot.

To elucidate dynamic changes of ETH-induced abscission of SDM shoots, the clustering affinity search technique (CAST) was employed to generate clusters^10^. CAST analyses of the 10,158 expressed genes revealed 20 major clusters, with gene numbers within clusters ranging from 2,429 to 192. The 21,764 differentially expressed genes were grouped into 20 clusters based on their temporal expression patterns (Fig. 5). Figure 5 showed gene expression heatmap of 20 major clusters and illustrated the major gene-expression patterns during ETH-induced abscission of SDM shoots. Among 20 clusters, different sets of unigenes were upregulated or downregulated at varied stages; i.e., clusters 3, 7, 8, and 9, during early response to ETH; clusters 4, 16 and 20, during late response to ETH; and cluster 12 and 14 in abscised shoots (Fig. 5).

### Profiling the early and late response to ETH in non-abscised SDM shoots

**Figure 6:**
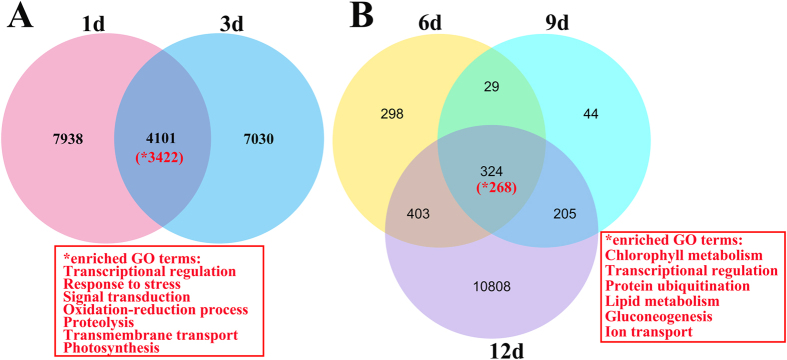
Differentially expressed unigenes and enriched GO terms in non-abscised SDM shoots. (**A** and **B**) Number of unigenes differentially expressed in non-abscised SDM shoots at early (**A**) and late stages (**B**) in response to ethephon. The enriched GO terms were represented by asterisk (*).

Early response (1 and 3 days) to ETH resulted in severe changes in gene expression. Differential expression showed that 12,040 and 11,131 unigenes were significantly differentially expressed at the early response, respectively (FDR < 0.05) (Fig. 6A; Supplemental Dataset 4). In addition, there were a total of 4,101 unigenes, which amounted for one-third of differentially expressed genes and showed overlap between both early time points, suggesting that the early steps of ETH response and signaling occur transiently (Supplemental Fig. S3). The GO analysis of this set upregulated genes revealed that a significant overrepresentation of genes involved in transcriptional regulation, response to stress, signal transduction, photosynthesis, oxidation-reduction process, proteolysis, and transmembrane transport (Fig. 6A). Interestingly, both of ethylene-responsive transcription factor (c49743_g1) and auxin-induced protein (c43642_g1) were more than two times up-regulated at the early stage. After 1 day of ETH treatment, several unigenes involved in hormone metabolism and regulation process were significantly upregulated (p values < 0.0007). At 3 days, ETH treatment led to significant increase in the number of differentially expressed genes. Upregulated unigenes were enriched in oxidoreductase oxido-reductase, and transcription activities.

Differential expression analysis of late response (6 days or more) showed that only a few unigenes (209) were commonly significantly expressed after 3 days in non-abscised SDM shoots. However, great changes in gene expression, with 9406 unigenes being significantly differentially expressed, occurred at 12 days after ETH treatment. It is also worth noting that the number of differentially expressed genes was the lowest at 9 days (Fig. 6B; Supplemental Fig. S4). GO analysis of this set genes revealed that a significant overrepresentation of genes was involved in chlorophyll metabolism, transcriptional regulation, protein ubiquitination, lipid metabolism, gluconeogenesis and ion transport (Fig. 6B). Interestingly, a histidine kinase (c56677_g1) was 90-fold upregulated during the late response. Some of the genes upregulated during the late response were overrepresented in the same gene categories. We also observed that unigenes upregulated were enriched in signal transduction, lipid metabolism, transcription regulation and transport functions. However, some unigenes up or down-regulated were not assigned to the known function.

### RNA-seq analysis of abscised shoots

**Figure 7:**
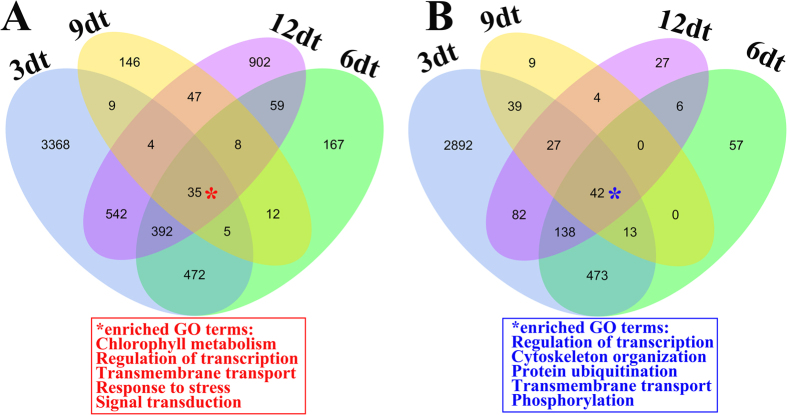
Overview of the transcriptional changes occurring in abscised SDM shoot induced by ethephon. Venn diagram representing unigenes upregulated (**A**) and downregulated (**B**) induced by ETH in abscised SDM shoots at 3, 6, 9, and 12 days. Red and blue asterisk (*) represented enriched GO terms of up- or down-regulated unigenes, respectively.

After ETH treatment, SDM shoots started to abscise at 3 days. We studied the transcriptomic behavior of the abscised shoots in detail. Comparing the transcriptomic data of abscised shoots collected at 3, 6, 9, and 12 days, a total of 77 unigenes were differentially expressed in all the abscised shoots (Fig. 7). Among them, 35 unigenes were upregulated, which were enriched in chlorophyll metabolism process, regulation of transcription, transmembrane transport, response to stress, and signal transduction. A total of 42 unigenes were downregulated in the abscised shots, with enriched GO terms including transcriptional regulation, cytoskeleton organization, protein ubiquitination, and transmembrane transport. Remarkably, we also found that the number of differentially expressed unigenes was the largest at 3 days, with 3,910 unigene upregulated and 3,089 downregulated (Supplemental Fig. S5; Supplemental Dataset 4). The number was minimum at 9 days, with a total of 326 unigenes differentially expressed. The results suggested that SDM shoots abscission induced by ETH has unique characteristics that are not well understood at the molecular level.

### Molecular basis of non-abscised and abscised shoots

To gain insight into the physiological and molecular factors underlying SDM shoot abscission, we analyzed the transcriptomic data of non-abscised and abscised shoots at 3 days after ETH treatment. The relationship between the two samples showed less correlation (average 0.65) than the average correlation (0.767) of each sample (Fig. 4), suggesting a clear distinction between the non-abscised and abscised shoots. We identified 9,817 and 1,314 unigenes were upregulated and downregulated at non-abscised shoots, and 3,910 and 3,089 unigenes were upregulated and downregulated at non-abscised shoots, respectively (Supplemental Fig. S6). Unigenes differentially expressed in abscised shoots were enriched in GO biological processes such as primary metabolism process, protein phosphorylation, lipid metabolism and small molecule metabolism, suggesting the importance of these metabolic pathways during ethylene regulation. On the other hand, unigenes differentially expressed in non-abscised shoots were enriched in GO biological processes such as transcriptional regulation, heterocycle metabolism, oxidation-reduction process, and regulation of macromolecule biosynthetic process. These results showed that the extensive metabolic changes occurred during shoot abscission induced by ETH.

**Figure 8:**
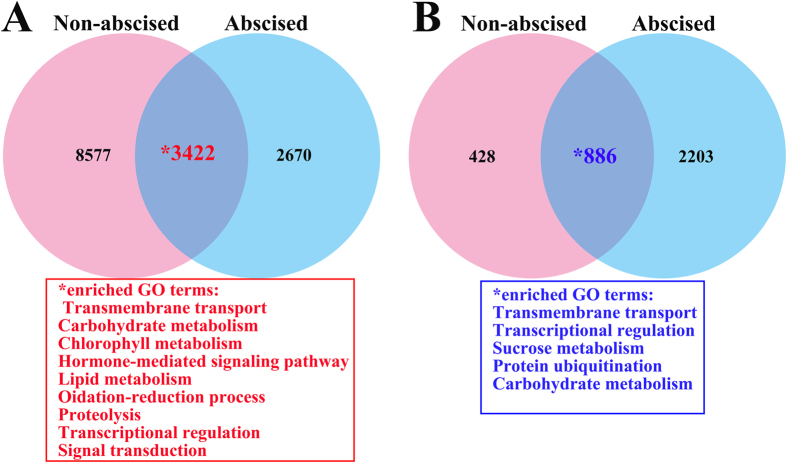
Comparative analysis of ethephon-responsive genes in non-abscised and abscised SDM shoots. (**A** and **B**) Number of unigenes up-regulated (**A**) and down-regulated (**B**) in non-abscised and abscised SDM shoot at 3 day after ETH treatment, respectively. The enriched GO terms were represented by asterisk.

We also noted that, of the 4,108 unigenes differentially expressed in both samples, 1,240 upregulated unigenes were enriched in GO terms as follows: transmembrane transport, carbohydrate metabolism, chlorophyll metabolism, hormone-mediated signaling pathway, lipid metabolism. GO is enriched for 886 downregulated unigenes implicated in transmembrane transport, transcriptional regulation, sucrose metabolismand carbohydrate metabolism (Fig. 8).

### Genes associated with SDM shoots response and abscission induced by ETH

**Figure 9:**
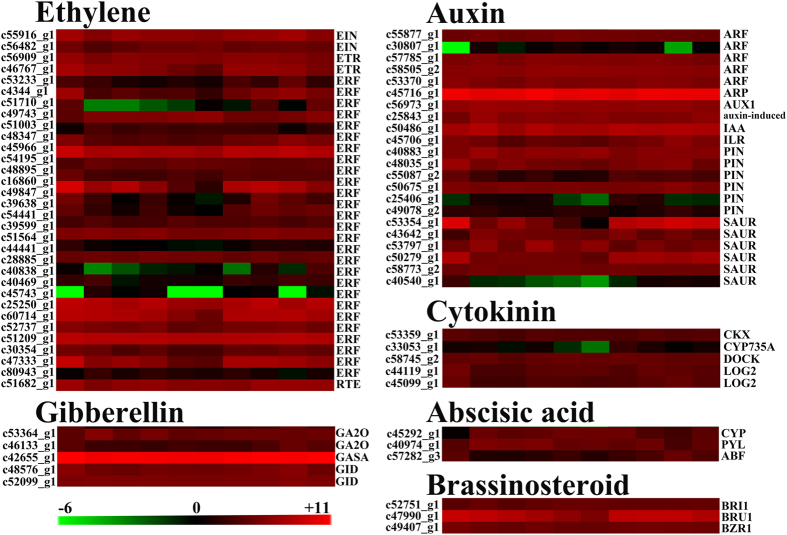
Expression profiles of phytohormone-associated genes in SDM shoots after ethephon treatment. Hierarchical clustering of the expression levels of differentially expressed hormone-associated genes including ethylene, abscisic acid, auxin, gibberellin, brassinosteroid, and cytokinin involved in biosynthesis, degradation and signaling during ETH response.

We analyzed in detail several classes of candidate genes associated with SDM abscission shoots. First, a total of 68 candidate genes were found related to phytohormone biosynthesis and signaling (Fig. 9; Supplemental Dataset 5), which were significantly differentially expressed (p < 0.05) during shoot abscission induced by ETH. Of these, 31, 21, 3, 5, 3, and 5 unigenes were associated with ethylene, auxin, ABA, GA, brassinosteroid, and cytokinin, respectively. These genes should be closely relevant with shoot abscission, including those encoding ethylene biosynthesis and transduction pathway (ERF), AP2/ERF transcription factor and ethylene receptor (ETR), auxin response factor (ARF), SAUR family protein, auxin efflux carrier component (PIN), auxin influx carrier (AUX1), gibberellin 20 oxidase (GA20), gibberellin receptor (GID1), cytokinin hydroxylase (CYP), and brassinosteroid signaling (BRZ). However, genes related to salicylic acid and jasmonic acid were not differentially expressed during shoot abscission. These results suggested that six of the eight classes of plant hormones were involved in the process of shoot abscission induced by ETH treatment. The most important hormones were ethylene and auxin, followed by GA, Cytokinin, Brassinosteroid, and ABA, according to the number of differentially expressed genes.

Transcription factors are major regulatory switches during development and stress. We identified transcription factors differentially expressed in response to ETH. A total of 512 annotated transcription factors showed differential expression (Fig. 9; Supplemental Dataset 5), including members of the AP2/ERF, bZIP, MADS, MYB, and WRKY families. Some more abundant transcription factors with previously reported roles in ethylene-responsive signaling were identified, such as OVM (c51003_g1) and ANT (c47333_g1). However, some of the most abundant differentially expressed transcription factors that could not be assigned to known families were annotated as hypothetical proteins. Upregulated transcription factors could be related with triggering the transcriptional reprogram during ETH-induced shoots abscission.

During SDM shoots abscission, we also identified 727 unigenes related to a transporter that were differentially expressed. Among them, 450 and 277 unigenes were up- and down- regulated at early response stage of ETH, respectively. These genes were overrepresented in transmembrane, amino acid, and sugar transporter, indicating that these genes represented an instantaneous response to the ETH treatment. For example, c113521_g1 (sugar transporter) was six times higher at an early stage than the control; c50624_g2 (calcium channel protein) and c45044_g1 (sucrose transport protein) showed strongly decreased expression during the early stage (Fig. 9). These results suggest that transporter may play regulatory roles in transport of ion and sugar and perception of signaling molecules.

Calcium has been considered as an important intracellular messenger in plants and is a key for structural integrity of cell wall. Carbohydrate metabolism is involved in abscission as well. A total of 78, 20, and 24 unigenes involved in carbohydrate metabolism, calcium signaling, and cell wall modification were found to be differentially expressed during shoots abscission, respectively (Fig. 9; Supplemental Dataset 5). Not surprisingly, ETH treatment also affected the carbohydrate and energy metabolism. For example, c13449_g1 (5′-AMP-activated protein kinase subunit), c9029_g1 (calmodulin) and c105396_g1 (PIR protein repeat) were significantly upregulated while c53357_g1 (pfkB family carbohydrate kinase) and c58097_g1 (calmodulin) were downregulated. This suggests the involvement of these genes in ETH-induced shoot abscission. Cell wall modification are critical for abscission in other plants. Here, we found at least 21 differentially expressed unigenes related to cell wall modification (Supplemental Fig. 7). Among then, 13 unigenes were significantly upregulated during abscission of shoot. The increase in the expression of these 13 genes associated with cell wall abscission is consistent with tomato flower abscission^11^. Furthermore, our anatomy showed programed cell death happen in the abscission zone. It is reported that programmed cell death is involved in abscission of tomato^12^. We identified 16 unigenes associated with cell death that were differentially expressed during shoot abscission (Supplemental Fig. 7). Most of genes were upregulated during shoot abscission of SDM induced by ETH.

## Discussion

Our goal was to identify genes involved in ethylene-dependent shoots abscission in *A. sichuanense* by examining genome-wide transcript changes in response to ethylene over time. In this study, a total 21,764 were differentially expressed during shoot abscission induced by ETH. Our results showed that the greatest number of unigenes with changed expression was found at the onset of abscission. In addition, the genes associated with ethylene and IAA biosynthesis and signaling were significantly upregulated at this time, indicating that the ethylene regulation of abscission may have a significant role. In this regard, the genes identified herein define pivotal processes associated with ETH-induced shoot abscission including cell wall hydrolysis and modification, lipid transference, hormone regulation, transport, transcriptional control, and oxidation-reduction process. Genome-wide identification of genes expressed in non-abscised and abscised shoots is the first step in elucidating the pathways and molecular mechanisms underlying ETH function controlling spruce dwarf mistletoe.

Transcriptomic profiles during ETH-induced shoot abscission suggested that the onset and progression of shoot abscission are accompanied by changes in expression of a large number of genes and activation of genesis required for the onset of abscission. Similarly, in mandarin fruit (*Citrus unshiu* Marc.), 1493 genes were identified as ethylene-responsive with more than 3-fold expression change at 72 h after ethylene treatment, and more than half of the ethylene-responsive genes were repressed, indicating that ethylene demotes numerous biological processes and plays an important role in fruit ripening and senescence^13^.

In other words, our data was consistent with mature citrus fruit abscission, which commenced with the activation of ethylene signal transduction pathway that led to the activation of ethylene-responsive transcription factors and the subsequent transcriptional regulation of a large set of ethylene responsive genes^14^. In soybean, ETH treatment significantly increased the abscission rate of flowers and pods. Strong correlations were observed among different gene expression profiles and specific metabolite groups^15^. Ethylene was shown to activates expression of genes encoding cell wall remodeling enzymes and their secretion to cell walls^16^,^17^,^18^. A large number of transcription factors and some putative signaling components were highly regulated by ethylene in leaves of *Arabidopsis thaliana*^19^. Previous study on Clementina de Nules (*Citrus clementina*) showed that ethylene regulated 725 of the 12, 672 cDNA probes printed in the microarray, 509 were preferentially expressed in the Pet and 216 in the LAZ-enriched tissues, and functional annotation of differentially expressed genes highlighted key processes regulating the activation and progress of the cell separation that brings about abscission. These included cell-wall modification, lipid transport, protein biosynthesis and degradation, and differential activation of signal transduction and transcription control pathways^20^. Ethylene-treated citrus leaf activated program dominated by the expression of genes associated with protein synthesis, protein fate, cell type differentiation, development and transcription^21^. The de novo transcriptome analysis of *Gardenia jasminoides* resulted in the identification of prevailing families of differentially expressed transcriptional factors with specific temporal expression patterns such as two WRKYs and one bHLH, which might play the role of senescence progression regulators^22^. These studies will contribute to a better understanding of the molecular regulatory mechanism of plant abscission induced by ETH.

Our data observed 68 candidate genes related to phytohormone biosynthesis and signaling were significantly differentially expressed during shoot abscission. Of the 68 differentially expressed unigenes, 31, 21, 3, 5, 3, and 5 unigenes were associated with ethylene, auxin, ABA, GA, brassinosteroid, and cytokinin, respectively. However, genes associated with salicylic acid and jasmonic acid were not identified during shoot abscission. These results suggest that ETH affects the transcriptional regulation of the above mentioned six classes of plant hormones in the process of shoot abscission induced by ETH. The most important hormones were ethylene and auxin, followed by GA, cytokinin, brassinosteroid and ABA, according to the number of differentially expressed genes. According to our results and previous findings, we demonstrated that the above-mentioned plant hormones were involved in the process of SDM shoot abscission induced by the ETH treatment, and the most important hormones were ethylene and auxin.

Transporters are involved in hormone perception or signaling^23^. In this study, we found that 727 unigenes encoding transporters were differentially expressed during SDM shoot abscission. The expression of biosynthesis, metabolism, and signaling for the above eight mentioned phytohormones was investigated in *Arabidopsis* by van der Graaff *et al*.^24^. They suggested that the expression profiles of the genes associated with hormone biosynthesis and signaling revealed additional players in the senescence regulatory network. Our findings support hypotheses that ethylene sensing and response genes possibly accelerate ETH-induced shoot abscission.

It should be noted that this study only focuses on the gene expression profile occurring in the shoot abscission induced by ETH. At 1 day after ETH treatment, unigenes involved in hormone metabolism and regulation process were significantly upregulated. At 3 days, the abscission of SDM shoots began and reaches the peak value at 9 days. Within 3 days, ETH induced the upregulation of genes functioning transcriptional regulation, response to stress, signal transduction, photosynthesis, oxidation-reduction process, proteolysis, and transmembrane transport. However, after 3 days up to 12 days, the genes associated with chlorophyll metabolism, transcriptional regulation, protein ubiquitination, lipid metabolism, gluconeogenesis and ion transport were upregulated. In the abscised shoots, differentially expressed genes were enriched in chlorophyll metabolism process, regulation of transcription, transmembrane transport, response to stress, and signal transduction while unigenes downregulated in the abscised shots, with enriched GO terms including transcriptional regulation, cytoskeleton organization, protein ubiquitination, and transmembrane transport. During ETH treatment, the most affected genes included those related to ethylene biosynthesis and signaling, transcription factors, transporters, carbohydrate etc. Then, the cell separation occurred, and the shoot abscission was enhanced.

In summary, this study depicts the transcriptomic dynamics in the abscission of *A. sichuanense* shoots induced by ETH. To the best of our knowledge, this is the first report to monitor the gene expression profile occurring during SDM shoots abscission induced by ethephon on the genome-wide level. This study will contribute to the better understanding of the molecular regulatory mechanism of shoots abscission in SDM.

## Materials and Methods

### Experimental site and ETH treatment

The experimental site, Xianmi Forest Farm, is located in northeastern of Qinghai province, China, and covers an area of 0.187 M ha. The study site is 25 ha in size and is a pure natural Qinghai spruces stand that comprised of mature and young spruce trees infected by SDM. In 2014, we randomly selected six plots and chose 18 infected spruces, which were sprayed with 200 or 400 ppm concentrations of ETH. After ETH application, we surveyed the SDM survival every day until 12 days. Additional control plants were sprayed with distilled water. The experiment was replicated three times. The non-abscised shoots of SDM were collected at 1, 3, 6, 9 and 12 days after 200 ppm ETH application. Since the abscission occurred at 3 days after ETH application, the abscised shoots were collected at 3, 6, 9, and 12 days by shaking the spruce branches to harvest the fresh abscised shoots. The plant samples were quickly frozen in liquid nitrogen and stored at −80 °C and later used for RNA extraction.

### RNA extraction and RNA-Seq sequencing

Total RNA was isolated from shoots from each sampled time point using Guanidine thiocyanate-Chloroform method and treated with DNA-free™ DNA Removal Kit (Ambion). RNA purity was checked using the NanoPhotometer spectrophotometer (IMPLEN, USA). Before cDNA synthesis, RNA concentration was measured using Qubit RNA Assay kit (Life Technologies).

First, the total RNA of each sample was equally pooled to prepare for the de novo assembly of SDM transcriptome. Secondly, about 2 μg of total RNA per sample was used as input material for the digital gene expression (DGE) preparations. DGE RNA-seq libraries were prepared from two biological replicates using a custom high-throughput method for the Illumina RNA-seq library^25^. These RNA-Seq libraries were sequenced on an Illumina Hiseq 2000 platform at Novogene Bioinformatics Technology Co., Ltd. (Beijing, China), and 100 bp paired-end reads were generated.

### De novo assembly transcriptome and functional annotation

Raw sequenced reads were processed using Trimmomatic software^26^, and clean reads were obtained by removing reads containing adapter, reads containing ploy-N, and low quality reads from raw data. All the downstream analyses were based on clean reads with high quality. All the resultant filtered and trimmed set of high-quality reads for each library was then de novo assembled using the Trinity software package^27^. To calculate abundance estimation for each unigene, clean reads were mapped back onto the assembled transcriptome, and the read count for each unigene was obtained from the mapping results by RSEM^28^. We used the term FPKM (fragments per kilobase per transcript per million mapped reads) to estimate gene expression abundance. Unigene with one or more FPKM were retained for downstream analysis.

The assembled unigene from the final transcriptome was annotated, aligned, and compared with the NCBI non-redundant (NR) database, NCBI nucleotide sequences (NT) database, eukaryotic ortholog groups (KOG) database, and KEGG ortholog (KO) database, respectively, using BLASTX with a significance threshold of E-value ≤ 10^−5^. Unigenes were also compared against the UniProt database and protein family (PFAM) database using default parameters.

The GO terms describing biological processes, molecular functions, and cellular components for functional categorization were analyzed using Blast2go software^29^. The E-value filter for GO annotation was 1e^−6^. The pathway assignments were carried out by sequence searches against the KEGG database, also using the BLASTX algorithm with an E-value threshold of 10^−5^. After the processes, proper GO terms and Enzyme Code numbers from the KEGG pathway were generated.

### Read mapping and gene expression analysis

All clean reads were mapped back to the above-assembled transcriptome using RSEM software. Read count for each unigene was obtained from the mapping results, and FPKM were estimated by RSEM. For gene expression analysis, differential expression analysis of each condition was performed using the DESeq R package^30^. Twenty paired-libraries including AsCk_1, AsCk_2, As1d_1, As1d_2, As2d_1, As2d_2, As3d_1, As3d_2, As6d_1, As6d_2, As9d_1, As9d_2, As12d_1, As12d_2, As3dt_1, As3dt_2, As6dt_1, As6dt_2, As9dt_1, As9dt_2, As12dt_1, and As12dt_2 (Table 1) were used to analyze the differential gene expression. DESeq provides statistical routines for determining differential expression in digital gene expression data using a model based on the negative binomial distribution. The resulting P values were adjusted using the Benjamini and Hochberg’s approach for controlling the false discovery rate. Genes with an adjusted P-value (FDR < 0.05) were assigned as differentially expressed using the edgeR software package 31.

A MultiExperiment Viewer^10^ was used to visualize changes in gene expression. Pearson correlation coefficient was calculated from the six samples according to genes’ expression profiles. Venn diagrams were drawn through the interactive tool of VENNY^32^.

### Protein measurements

For protein measurement, 200 μg of individual shoot samples was ground in liquid nitrogen, and then the powder was mixed with plant total protein extraction buffer (Applygen Technologies Inc., Beijing, China) and centrifuged (10 000 × g, 10 min) at 4 °C. Protein crude extract (3 mL) was re-extracted according to the manufacturer’s instructions. Total proteins were quantified by adding it to the extraction solution of the kit P1511. The average expression values of the three biological replicates for each time point was generated.

### Hormone measurement

The hormones indole acetic acid (IAA), abscisic acid (ABA), gibberellic acid (GA), and salicylic acid (SA) were measured in individual samples. The shoots were ground in liquid nitrogen, and then phytohormones were extracted from 50 mg tissue (fresh weight) of three biological replicates using 500 ml extraction solution (2-propanol/H_2_O/concentrated HCl = 2:1:0.002, vol/vol/vol) and high-performance liquid chromatography–mass spectrometry (HLPC-MS) method as described by Pan *et al*.^33^. The tubes were kept on a shaker at a speed of 100 rpm at 4 °C for 30 min, 1 ml dichloromethane was added and then shaken for another 30 min. The mixture was centrifuged at 13,000 rpm at 4 °C for 5 min, and approximately 900 μl of lower phase was transferred to a new tube. The sample solutions (50 μl) were injected into HLPC (Agilent Technologies, Santa Clara, CA USA)-MS (AB SCIEX Deutschland GmbH, Darmstadt, Germany) system to detect the four phytohormones contents, respectively.

### Microscopic observation of SMD shoots colonizing in the spruce branches

The spruce branches colonized by SMD were collected at 6 days after after ETH application. The structure of SMD shoots colonizing in the spruce branches were observed with paraffin sectioning and light microscope method as described by Zhu *et al*.^34^. Samples were first fixed with FAA solution (10 ml formaldehyde, 50 ml alcohol and 5 ml glacial acetic acid in 100 ml water) and embedded in wax. Then, they were sectioned into slices of 12 μM thickness by Leica RM2235 (Germany). After being dewaxed and stained with a sarranine-fast green solution, the slices were discolored with alcohol and observed under light microscope (Leica DM1000, Germany).

### Availability of sequencing data

Raw Illumina sequences were deposited inNCBI SRA (http://trace.ncbi.nlm.nih.gov/Traces/sra/) under accession SRR3085214 for the all mix samples, the accession number from SRR3085388 to SRR3085410 for the SDM shoots after ETH application and water as a control.

## Additional Information

**How to cite this article**: Wang, Y. *et al*. High-resolution transcript profiling reveals shoot abscission process of spruce dwarf mistletoe *Arceuthobium sichuanense* in response to ethephon. *Sci. Rep.* **6**, 38889; doi: 10.1038/srep38889 (2016).

**Publisher's note:** Springer Nature remains neutral with regard to jurisdictional claims in published maps and institutional affiliations.

## Supplementary Material

Supplementary Information

Supplementary Dataset 1

Supplementary Dataset 2

Supplementary Dataset 3

Supplementary Dataset 4

Supplementary Dataset 5

## Figures and Tables

**Table 1:** Statistics of digital transcript abundance library sequencing and mapping.

Samples^a^	Raw reads	Clean reads^b^	Q20(%)^c^	Q30(%)^d^	Mapped reads
AsMix^e^	90823070	88944462	96.89	93.68	73607692^f^ (82.76%)^g^
AsCk_1	12671141	12421247	98.90	97.82	11470583 (92.35%)
AsCk_2	14066524	13859597	98.73	97.49	12710682 (91.71%)
As1d_1	14221021	13994898	98.68	97.43	12862767 (91.91%)
As1d_2	12294854	12014036	98.92	97.86	10981072 (91.40%)
As2d_1	12988889	12543584	98.74	97.55	11447616 (91.26%)
As2d_2	13457937	13185770	98.74	97.53	11730741 (88.97%)
As3d_1	14306331	14103709	98.69	97.44	12600297 (89.34%)
As3d_2	13797549	13361284	98.76	97.58	12129582 (90.78%)
As6d_1	11312526	11197276	97.87	95.93	9986860 (89.19%)
As6d_2	14071288	13993103	98.06	96.28	12647954 (90.39%)
As9d_1	15448107	15094827	98.07	96.29	13361422 (88.52%)
As9d_2	14207987	14011774	98.07	96.28	12248328 (87.41%)
As12d_1	13115631	12904421	98.40	96.88	11862696 (91.93%)
As12d_2	12900413	12774666	98.40	96.88	11690067 (91.51%)
As3dt_1	13527051	13290327	98.78	97.59	12017974 (90.43%)
As3dt_2	13064645	12780787	98.87	97.77	11690307 (91.47%)
As6dt_1	14163383	14011087	98.29	96.70	12721794 (90.80%)
As6dt_2	10273094	10104323	98.33	96.77	9268957 (91.73%)
As9dt_1	11758997	11705074	98.02	96.22	10336786 (88.31%)
As9dt_2	12417530	11799692	98.03	96.21	10556686 (89.47%)

^a^1 and 2 represent two independent biological replicates.

^b^The number of reads generated from sequencing after filtering low quality reads (Q≤5).

^c^Q20: The percentage of bases with a Phred value >20.

^d^Q30: The percentage of bases with a Phred value >30.

^e^All samples mixed as AsMix to generate the reference transcriptome.

^f^The number of reads from clean data that were mapped back onto the assembled transcriptome.

^g^The percentages of reads account for the mapped reads.

**Table 2:** De novo Assembly statistics for *A. sichuanense* transcriptome.

	Transcripts	Unigenes
Total number	166025	120316
Average length (bp)	764	619
Minimum length (bp)	201	201
Maximum length (bp)	31907	31907
Number, ≤500 bp	93974	80808
Number, >500 bp	72051	39508
N50^a^ (bp)	1287	973
N90^b^ (bp)	293	252
Total nucleotides	126801634	74493258

^a^N50 is defined as the length of the largest contig from all the contigs ranked smallest to largest that represents 50% of the assembly lengthy.

^b^N90 is defined as the length of the smallest transcript in the sorted list of all transcripts where the cumulative length from the largest transcript to the smallest transcript is at least 90% of the total length.

**Table 3:** Statistics of annotation results for *A. sichuanense* unigenes.

NR	NT	KO	UniProt	Pfam	GO	KOG	All database	>1 database
56487	13039	17536	34300	37550	39470	21512	6064	62736

NR, NCBI nonredundant database;

NT, NCBI nucleotide sequences database;

KO, KEGG Ortholog database;

UniProt, Swiss-Prot protein database;

Pfam, protein family database;

GO, Gene Ontology;

KOG, eukaryotic ortholog groups database.

## References

[b1] Hawksworth F. & Wiens D. Dwarf mistletoes: Biology, pathology, and systematics. (Agricultural Handbook 709. Washington, DC: USDA Forest Service, 1996).

[b2] Nickrent D. L., Garcia M. A., Martin M. P. & Mathiasen R. L. A phylogeny of all species of *Arceuthobium* (Viscaceae) using nuclear and chloroplast DNA sequences. Am J Bot 91, 125–138 (2004). 21653369 10.3732/ajb.91.1.125

[b3] Zhou Z. Studies on the biological characters and control Arceuthobium sichuanenese in Sanjiangyuan Forest Protection Master thesis (2007).

[b4] Xia B. . The effects of *Arceuthobium sichuanense* infection on needles and current-year shoots of mature and young Qinghai spruce (*Picea crassifolia*) trees. Forest Pathol 42, 330–337 (2012).

[b5] Worrall J. & Geils B. Dwarf mistletoes. The Plant Health Instructor. doi: 10.1094/PHI-I-2006-1117-01. (2006).

[b6] Wang Z., Gerstein M. & Snyder M. RNA-Seq: a revolutionary tool for transcriptomics. Nat Rev Genet 10, 57–63 (2009). 19015660 10.1038/nrg2484 PMC2949280

[b7] Ko S. . Transcriptome analysis of mistletoe (*Viscum album*) haustorium development. *Horticulture*, Environment, and Biotechnology 55, 352–361 (2014).

[b8] Ranjan A. . De novo assembly and characterization of the transcriptome of the parasitic weed dodder identifies genes associated with plant parasitism. Plant Physiol 166, 1186–1199 (2014). 24399359 10.1104/pp.113.234864 PMC4226353

[b9] Leslie T. & Baucom R. S. De novo assembly and annotation of the transcriptome of the agricultural weed *Ipomoea purpurea* uncovers gene expression changes associated with herbicide resistance. G3 (Bethesda) 4, 2035–2047 (2014). 25155274 10.1534/g3.114.013508 PMC4199709

[b10] Saeed A. I. . TM4: a free, open-source system for microarray data management and analysis. Biotechniques 34, 374–378 (2003). 12613259 10.2144/03342mt01

[b11] Meir S. . Microarray analysis of the abscission-related transcriptome in the tomato flower abscission zone in response to auxin depletion. Plant Physiol 154, 1929–1956 (2010). 20947671 10.1104/pp.110.160697 PMC2996037

[b12] Bar-Dror T. . Programmed cell death occurs asymmetrically during abscission in tomato. Plant Cell 23, 4146–4163 (2011). 22128123 10.1105/tpc.111.092494 PMC3246325

[b13] Fujii H. . Profiling ethylene-responsive genes in mature mandarin fruit using a citrus 22K oligoarray. Plant Sci 173, 340–348 (2007).

[b14] Cheng C., Zhang L., Yang X. & Zhong G. Profiling gene expression in citrus fruit calyx abscission zone (AZ-C) treated with ethylene. Mol Genet Genomics 290, 1991–2006 (2015). 25948248 10.1007/s00438-015-1054-2

[b15] Cheng Y. Q. . RNA-seq Analysis Reveals Ethylene-Mediated Reproductive Organ Development and Abscission in Soybean (*Glycine max* L. Merr.). Plant Mol Biol Rep 31, 607–619 (2013).

[b16] Sexton R. & Roberts J. Cell Biology of Abscission. Annu Rev Plant Physiol 33, 133–162 (1982).

[b17] Estornell L. H., Agusti J., Merelo P., Talon M. & Tadeo F. R. Elucidating mechanisms underlying organ abscission. Plant Sci 199-200, 48–60 (2013). 23265318 10.1016/j.plantsci.2012.10.008

[b18] del Campillo E. & Bennett A. B. Pedicel breakstrength and cellulase gene expression during tomato flower abscission. Plant Physiology 111, 813–820 (1996). 8754682 10.1104/pp.111.3.813 PMC157899

[b19] Zhong G. Y. & Burns J. K. Profiling ethylene-regulated gene expression in *Arabidopsis thaliana* by microarray analysis. Plant Mol Biol 53, 117–131 (2003). 14756311 10.1023/b:plan.0000009270.81977.ef

[b20] Agusti J., Merelo P., Cercos M., Tadeo F. R. & Talon M. Ethylene-induced differential gene expression during abscission of citrus leaves. J Exp Bot 59, 2717–2733 (2008). 18515267 10.1093/jxb/ern138 PMC2486473

[b21] Agusti J., Merelo P., Cercos M., Tadeo F. R. & Talon M. Comparative transcriptional survey between laser-microdissected cells from laminar abscission zone and petiolar cortical tissue during ethylene-promoted abscission in citrus leaves. BMC Plant Biol 9, 127 (2009). 19852773 10.1186/1471-2229-9-127 PMC2770498

[b22] Tsanakas G. F., Manioudaki M. E., Economou A. S. & Kalaitzis P. De novo transcriptome analysis of petal senescence in *Gardenia jasminoides* Ellis. BMC Genomics 15, 554 (2014). 24993183 10.1186/1471-2164-15-554 PMC4108791

[b23] Campbell E. J. . Pathogen-responsive expression of a putative ATP-binding cassette transporter gene conferring resistance to the diterpenoid sclareol is regulated by multiple defense signaling pathways in *Arabidopsis*. Plant Physiol 133, 1272–1284 (2003). 14526118 10.1104/pp.103.024182 PMC281622

[b24] van der Graaff E. . Transcription analysis of arabidopsis membrane transporters and hormone pathways during developmental and induced leaf senescence. Plant Physiol 141, 776–792 (2006). 16603661 10.1104/pp.106.079293 PMC1475451

[b25] Kumar R. . A High-Throughput Method for Illumina RNA-Seq Library Preparation. Front Plant Sci 3, 202 (2012). 22973283 10.3389/fpls.2012.00202 PMC3428589

[b26] Bolger A. M., Lohse M. & Usadel B. Trimmomatic: a flexible trimmer for Illumina sequence data. Bioinformatics 30, 2114–2120 (2014). 24695404 10.1093/bioinformatics/btu170 PMC4103590

[b27] Grabherr M. G. . Full-length transcriptome assembly from RNA-Seq data without a reference genome. Nat Biotechnol 29, 644–652 (2011). 21572440 10.1038/nbt.1883 PMC3571712

[b28] Li B. & Dewey C. N. RSEM: accurate transcript quantification from RNA-Seq data with or without a reference genome. BMC Bioinformatics 12, 323 (2011). 21816040 10.1186/1471-2105-12-323 PMC3163565

[b29] Conesa A. & Gotz S. Blast2GO: A comprehensive suite for functional analysis in plant genomics. Int J Plant Genomics 2008, 619832 (2008). 18483572 10.1155/2008/619832 PMC2375974

[b30] Wang L., Feng Z., Wang X., Wang X. & Zhang X. DEGseq: an R package for identifying differentially expressed genes from RNA-seq data. Bioinformatics 26, 136–138 (2010). 19855105 10.1093/bioinformatics/btp612

[b31] Robinson M. D., McCarthy D. J. & Smyth G. K. edgeR: a Bioconductor package for differential expression analysis of digital gene expression data. Bioinformatics 26, 139–140 (2010). 19910308 10.1093/bioinformatics/btp616 PMC2796818

[b32] Oliveros J. C. VENNY. An interactive tool for comparing lists with Venn Diagrams. http://bioinfogp.cnb.csic.es/tools/venny/index.html (2007).

[b33] Pan X., Welti R. & Wang X. Quantitative analysis of major plant hormones in crude plant extracts by high-performance liquid chromatography-mass spectrometry. Nat Protoc 5, 986–992 (2010). 20448544 10.1038/nprot.2010.37

[b34] Zhu N. . Anatomical Study on Endophytic System of Dwarf Mistletoe (*Arceuthobium sichuanense*). Acta Bot Boreal Occident Sin 35, 1342–1348 (2015).

