# Supplementary material for: High-resolution transcript profiling reveals shoot abscission process of spruce dwarf mistletoe Arceuthobium sichuanense in response to ethephon
Source: Sci Rep. 2016 Dec 12;6:38889. doi: 10.1038/srep38889 (PMC5151019; doi:10.1038/srep38889)
Supplement: Supplementary Information [file srep38889-s1.pdf]

High-resolution transcript profiling reveals shoot abscission process of spruce dwarf mistletoe *Arceuthobium sichuanense* in response to ethephon

Yonglin Wang[1], Dianguang Xiong[1], Ning Jiang[1], Xuewu Li[1,2], Qiqing Yang[3], Chengming Tian[1*]

[1] The Key Laboratory for Silviculture and Conservation of Ministry of Education, College of Forestry, Beijing Forestry University, Beijing, China

[2] Academy of Forest Inventory and Planning, State Forestry Administration, Beijing, China

[3] Forest Pest Control and Quarantine Station of Qinghai Province, Xining, China

Corresponding author: C. Tian, chengmt@bjfu.edu.cn, +861062336073

Supplementary Information

Supplemental Fig. S1 Unigene and transcript size distribution.

Unigene and transcript size distribution showing the high proportion of small-sized transcripts in the SDM assembled transcriptome

Supplemental Figure 2. Proportions of unigenes with different expression levels in the assembled transcriptome.

All samples with two biological duplicates (-1 and -2) in different time points of ASM shoots were presented. The percentages were calculated based on the FPKM intervals.

Supplemental Figure 3. Heatmap of differentially expressed unigenes at early response to ETH.

Heatmap represented differentially expressed unigenes at 1 and 3d. Heat-map values represent log-transformed relative expression values.

Supplemental Figure 4. Heatmap of differentially expressed unigenes at late response to ETH.

Heatmap represented differentially expressed unigenes at 6, 9, and 12 d. Heat-map values represent log-transformed relative expression values.

Supplemental Figure 5. Heatmap of differentially expressed unigenes in abscised shoots induced by ETH.

Heatmap represented differentially expressed unigenes in abscised shoots at 3, 6, 9, and 12 d. Heat-map values represent log-transformed relative expression values.

Supplemental Figure 6. Heatmap of differentially expressed unigenes in abscised and non-abscised shoots induced by ETH.

Heatmap represented differentially expressed unigenes in abscised and non-abscised shoots at 3 d. Heat-map values represent log-transformed relative expression values.

Supplemental Figure 7. Effects of shoot abscission on the differentially expressed unigenes associated with cell wall modification and programed cell death in abscised and non-abscised shoots induced by ETH.

Heatmap represented differentially expressed unigenes associated with cell wall modification (A) and programed cell death (B) of SDM shoot induced by ETH. Heat-map values represent log-transformed relative expression values.

Supplemental Dataset 1. Annotation of Unigenes form SDM final transcriptome.

Column titles: (1) Unigene id, (2) length of unigene, (3) NR Evalue, (4) NR Description, (5) NT Evalue, (6) NT Description, (7) KO Description, (8) Swissprot Evalue, (9) Swissprot Description, (10) PFAM description, (11) GO Biological Pathway, (12) BP Description , (13) GO Molecular Function, (14) MF Description, (15) GO Cellular Component, (15) CC Description, (16) KOG Description

Supplementary Dataset 2. GO data.

Number of annotated transcripts in each GO-category along with GO-level, score and parent GO-terms for each category under biological process, molecular function and cellular component.

Supplemental Dataset 3. Enzyme code distribution from KEGG for all annotated SDM transcripts.

Column titles: (1) Pathway Hierarchy1, (2) Pathway Hierarchy1, (3) KEGG Pathway, (4) KEGG Pathway ID, (5) Number of annotated unigenes in the class, (6) List of unigenes.

Supplemental Dataset 4. Differentially expressed unigenes (P adjusted value < 0.05) for each pair-wise comparison for all samples.

Pair-wise comparisons are following: 1dvsCk, 3dvsCk, 6dvsCk, 9dvsCk, 12dvsCk, 3dtvsCk, 6dtvsCk, 9dtvsCk, 12dtvsCk, and 3dtvs3d.

Supplemental Dataset 5. Expression levels and annotation of selected multiple gene families differentially expressed in SDM shoots induced by ETH.

Gene families are as follows: phytohormone-associated genes, calcium signaling, carbohydrate metabolism, photosynthesis, transcription factors, and transporters.

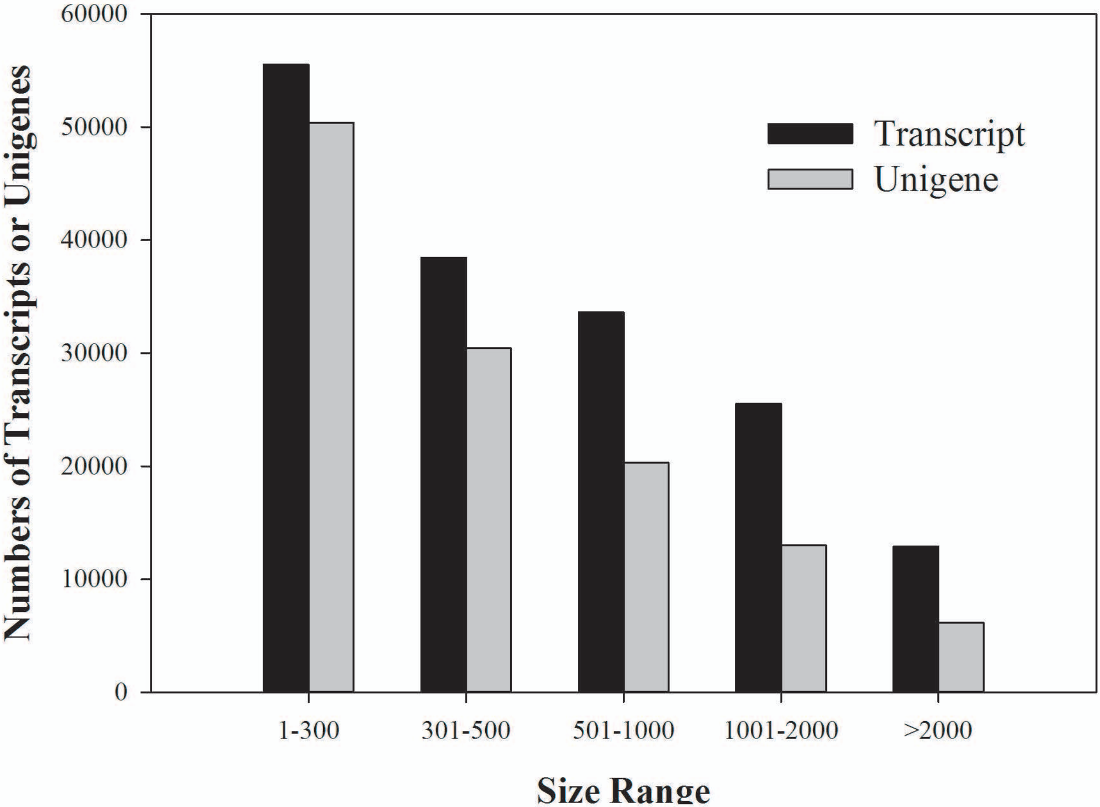
Numbers of Transcripts or Unigenes
60000
50000
40000
30000
20000
10000
0
Transcript
Unigene
1-300
301-500
501-1000
1001-2000
>2000
Size Range

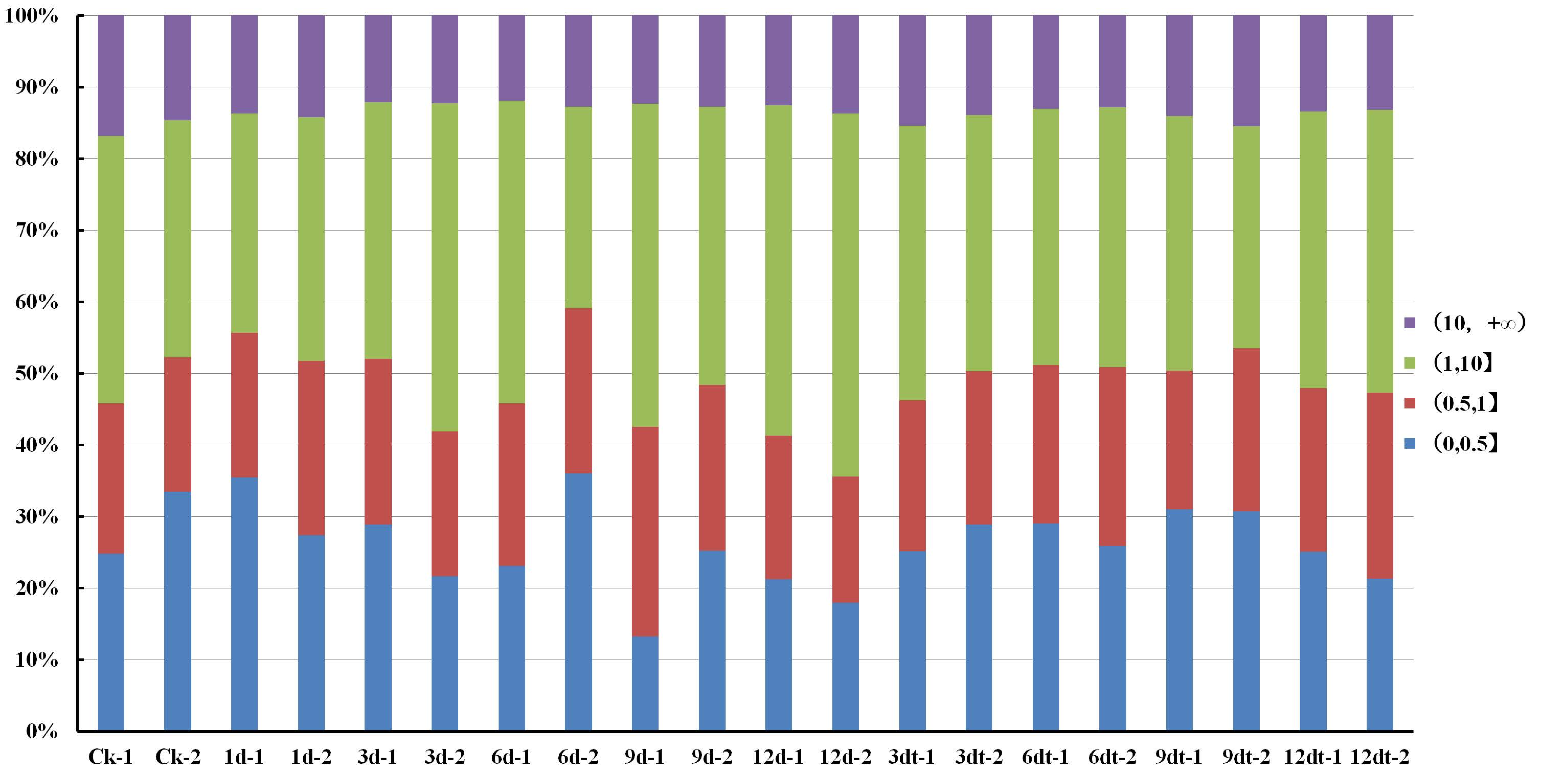

100%
90%
80%
70%
60%
50%
40%
30%
20%
10%
0%
Ck-1
Ck-2
1d-1
1d-2
3d-1
3d-2
6d-1
6d-2
9d-1
9d-2
12d-1
12d-2
3dt-1
3dt-2
6dt-1
6dt-2
9dt-1
9dt-2
12dt-1
12dt-2
（10，+∞）
（1,10】
（0.5,1】
（0,0.5】

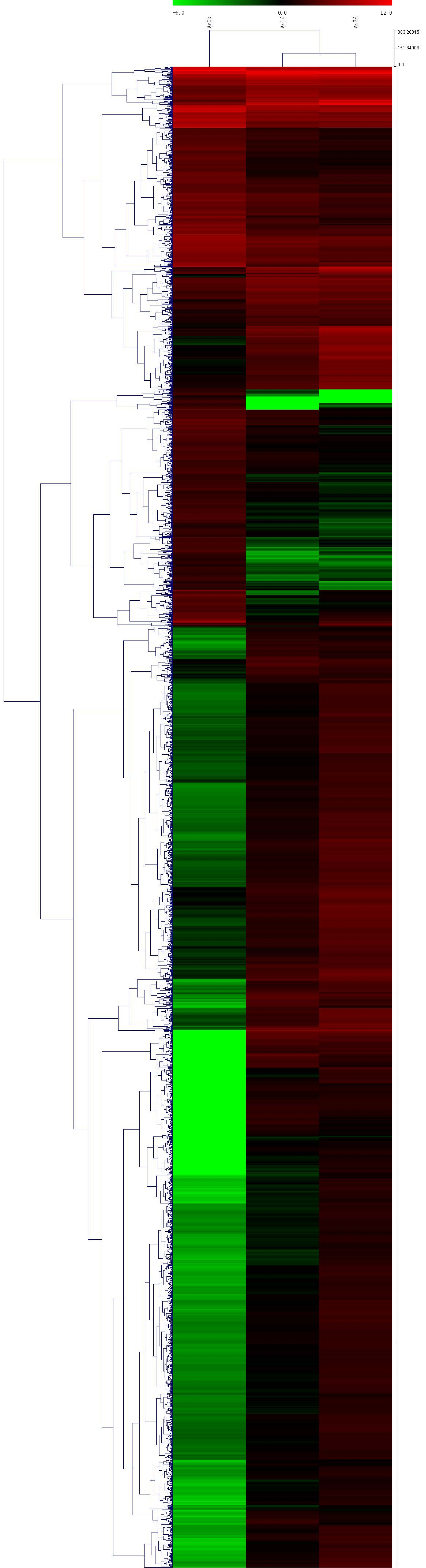
-6.0
0.0
12.0
AsCk
As1d
As3d
303.28015
151.64008
0.0

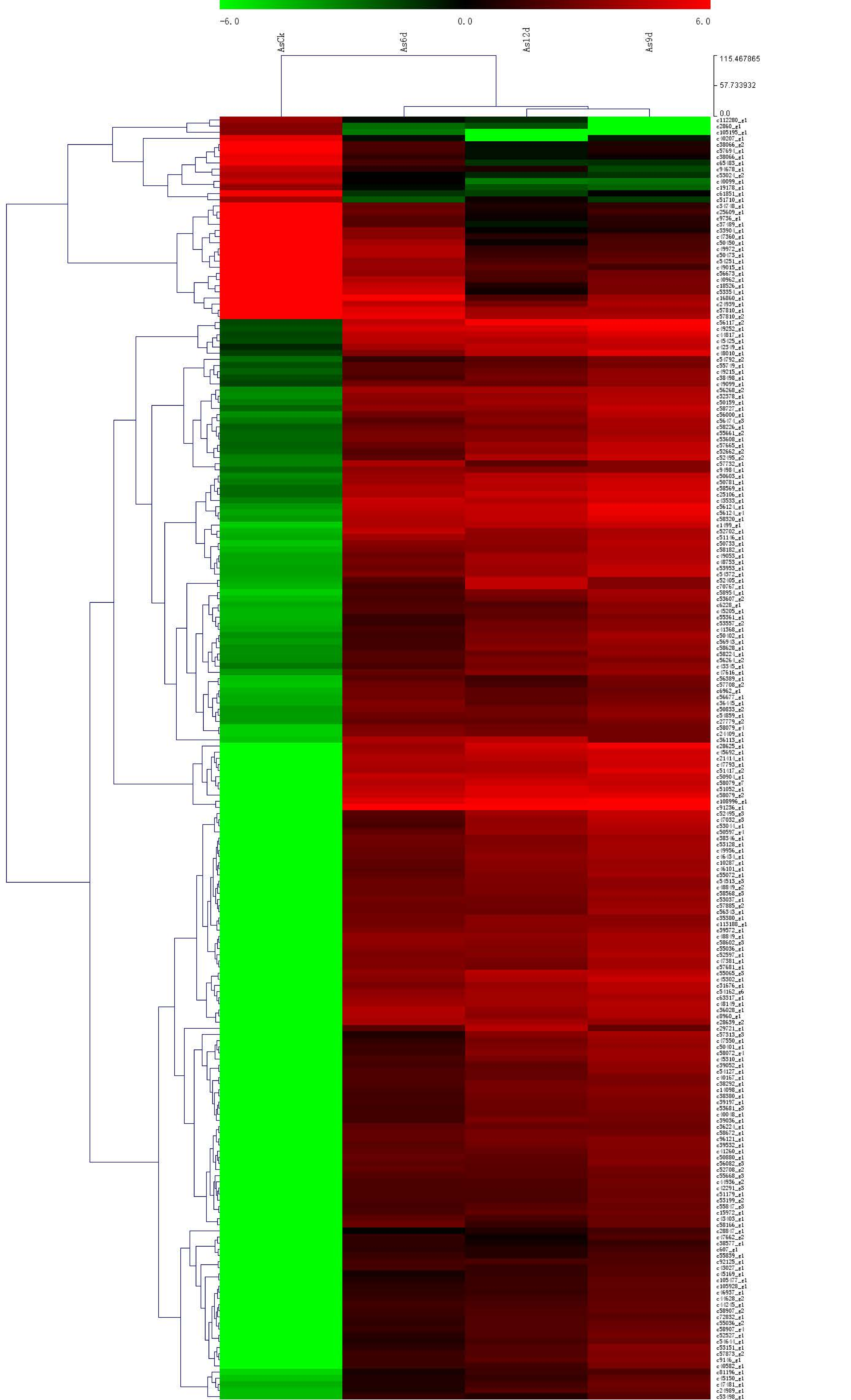

-6.0
0.0
6.0
AsCk
As6d
As12d
As9d
115.467865
57.733932
0.0

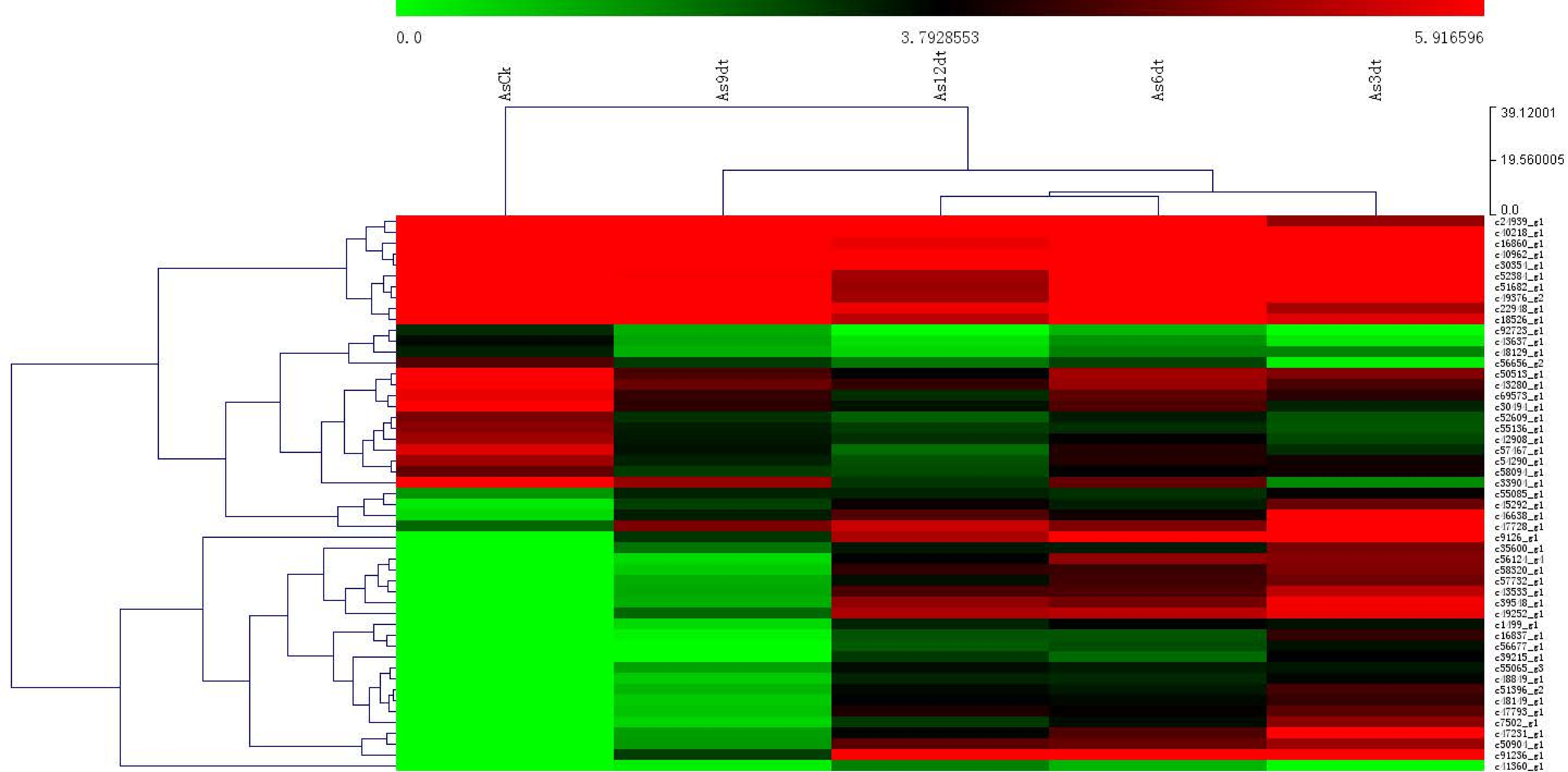

0.0
3.7928553
5.916596
AsCk
As9dt
As12dt
As6dt
As3dt
39.12001
19.560005
0.0
c24939_g1
c40218_g1
c16860_g1
c40962_g1
c30354_g1
c52384_g1
c51682_g1
c49376_g2
c22948_g1
c18526_g1
c92723_g1
c43637_g1
c48129_g1
c56656_g2
c50513_g1
c43280_g1
c69573_g1
c30494_g1
c52609_g1
c55136_g1
c42908_g1
c57467_g1
c54290_g1
c58094_g1
c33904_g1
c55085_g1
c45292_g1
c46638_g1
c47728_g1
c9126_g1
c35600_g1
c56124_g4
c58320_g1
c57732_g1
c43533_g1
c39548_g1
c49252_g1
c1499_g1
c16837_g1
c56677_g1
c39215_g1
c55065_g3
c48849_g1
c51396_g2
c48149_g1
c47793_g1
c7502_g1
c47231_g1
c50904_g1
c91236_g1
c41360_g1

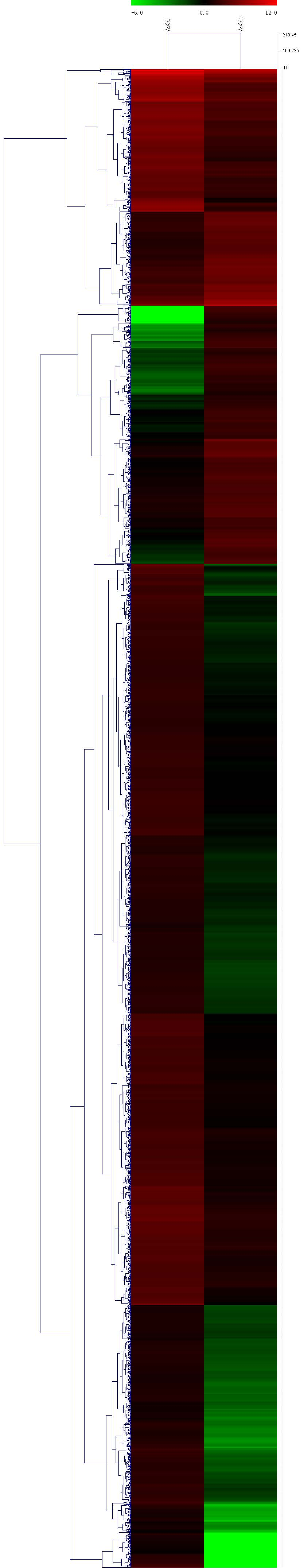

-6.0
0.0
12.0
As3d
As3dt
218.45
109.225
0.0

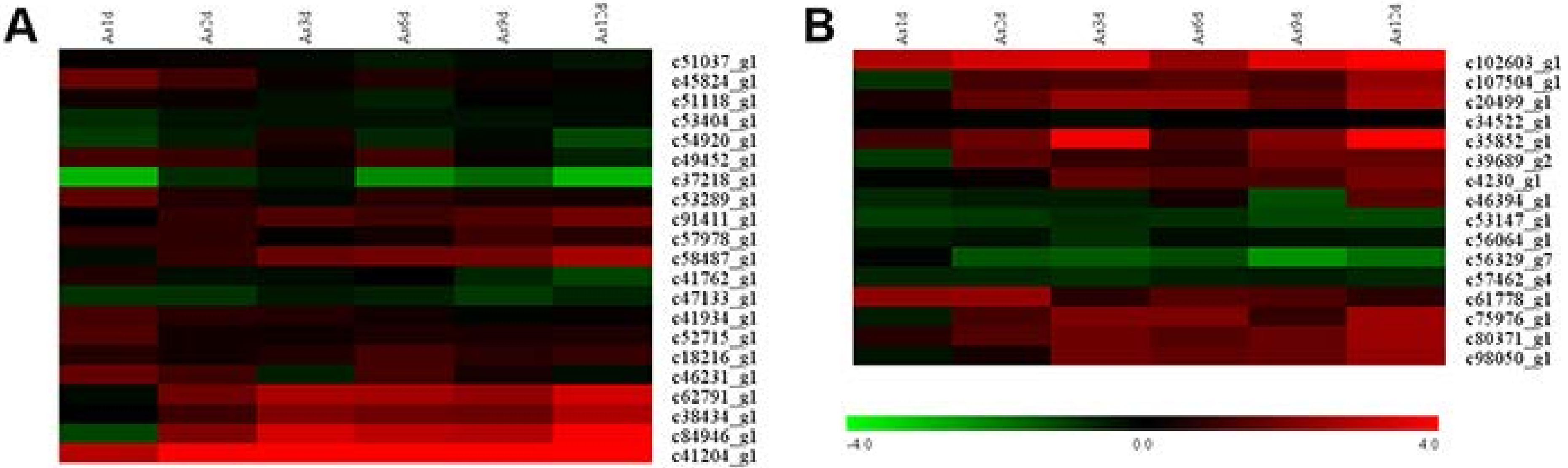
A
c51037_g1
c45824_g1
c51118_g1
c53404_g1
c54920_g1
c49452_g1
c37218_g1
c53289_g1
c91411_g1
c57978_g1
c58487_g1
c41762_g1
c47133_g1
c41934_g1
c52715_g1
c18216_g1
c46231_g1
c62791_g1
c38434_g1
c84946_g1
c41204_g1
B
c102603_g1
c107504_g1
c20499_g1
c34522_g1
c35852_g1
c39689_g2
c4230_g1
c46394_g1
c53147_g1
c56064_g1
c56329_g7
c57462_g4
c61778_g1
c75976_g1
c80371_g1
c98050_g1
-4.0
0.0
4.0
